# Supplementary material for: CTH/H2S Regulates LPS-Induced Inflammation through IL-8 Signaling in MAC-T Cells
Source: Int J Mol Sci. 2022 Oct 5;23(19):11822. doi: 10.3390/ijms231911822 (PMC9570289; doi:10.3390/ijms231911822)
Supplement: Supplementary file 1 [file ijms-23-11822-s001.zip › housekeeping gene selection.pdf]

1. Regarding the reason for choosing *GAPDH* as an internal reference:

GAPDH gene has a highly conserved sequence, its expression level in MAC-T cells is relatively constant, and it is not affected by the inclusion of some recognition sites and other inducing substances and maintains stable expression. Therefore, it was used as an internal reference gene in qPCR to normalize mRNA levels to *GAPDH* RNA transcript levels present in the same sample. The following is a small part of the qPCR experimental data and the agarose gel electrophoresis of the PCR products involved in this study. It can be seen that the expression of *GAPDH* is basically stable, consistent with the expected product size and has no heterobands and dimers, indicating its specificity better.

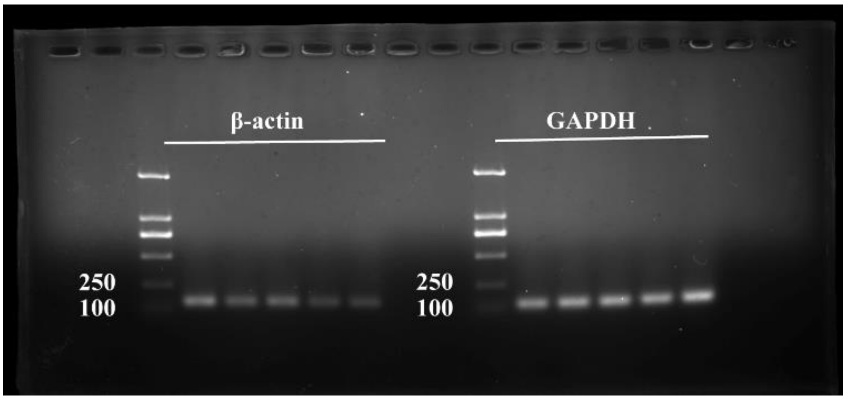

Fig.5			Fig.4			Fig.3		
Sample name	Gene	Cq	Sample name	Gene	Cq	Sample name	Gene	Cq
NC	GAPDH	17.90	NC	GAPDH	15.66	NC	GAPDH	15.79
NC	GAPDH	17.93	NC	GAPDH	15.56	NC	GAPDH	15.71
NC	GAPDH	17.85	NC	GAPDH	15.61	NC	GAPDH	15.78
LPS	GAPDH	18.05	LPS	GAPDH	15.31	LPS	GAPDH	15.49
LPS	GAPDH	18.16	LPS	GAPDH	15.27	LPS	GAPDH	15.55
LPS	GAPDH	18.06	LPS	GAPDH	15.37	LPS	GAPDH	15.43
LPS+SB	GAPDH	17.84	LPS+BCA	GAPDH	15.54	LPS+BCA	GAPDH	15.42
LPS+SB	GAPDH	17.88	LPS+BCA	GAPDH	15.46	LPS+BCA	GAPDH	15.37
LPS+SB	GAPDH	17.85	LPS+BCA	GAPDH	15.58	LPS+BCA	GAPDH	15.41
LPS+SB+NaHS	GAPDH	17.71	LPS+BCA+NaH	GAPDH	15.44	LPS+BCA+NaHS	GAPDH	15.59
LPS+SB+NaHS	GAPDH	17.74	LPS+BCA+NaH	GAPDH	15.35	LPS+BCA+NaHS	GAPDH	15.47
LPS+SB+NaHS	GAPDH	17.72	LPS+BCA+NaH	GAPDH	15.47	LPS+BCA+NaHS	GAPDH	15.52
LPS+NaHS	GAPDH	17.93	LPS+NaHS	GAPDH	15.33	LPS+NaHS	GAPDH	15.80
LPS+NaHS	GAPDH	17.95	LPS+NaHS	GAPDH	15.35	LPS+NaHS	GAPDH	15.78
LPS+NaHS	GAPDH	17.92	LPS+NaHS	GAPDH	15.40	LPS+NaHS	GAPDH	15.81

2.Primer specificity detection:

By Blast alignment results, there were no non-specific results, combined with melting curve and gel results, indicating better primer specificity .

Primer pair 1

	Sequence (5'->3')	Length	Tm	GC%	Self complementarity	Self 3' complementarity
Forward primer	GGTCACCAGGGCTGCTTT	18	59.88	61.11	5.00	1.00
Reverse primer	CTGTGCCGTTGAACTTGC	18	57.39	55.56	5.00	3.00

Products on target templates

>NM_001034034.2 Bos taurus glyceraldehyde-3-phosphate dehydrogenase (GAPDH), mRNA

```
product length = 128
Forward primer  1    GGTCACCAGGGCTGCTTT  18
Template        116  ..................  133

Reverse primer  1    CTGTGCCGTTGAACTTGC  18
Template        243  ..................  226
```

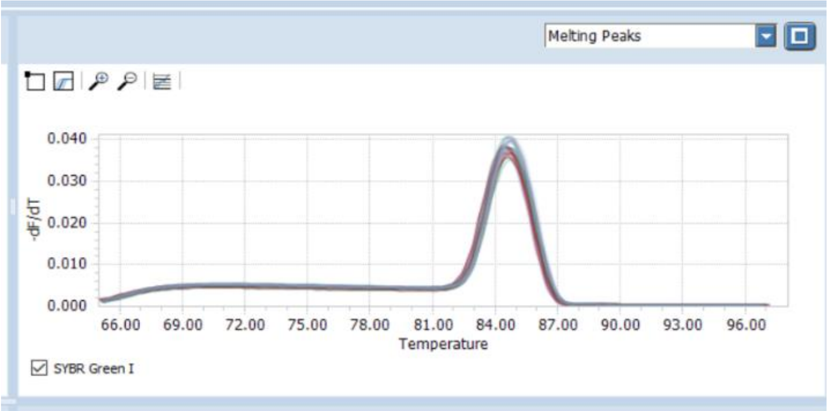

3. Primers used in this study to span introns:

The upstream primer across the intron is on one exon, and the downstream primer is on the other exon. According to the detection, the reference gene primer used in this study is across the intron.

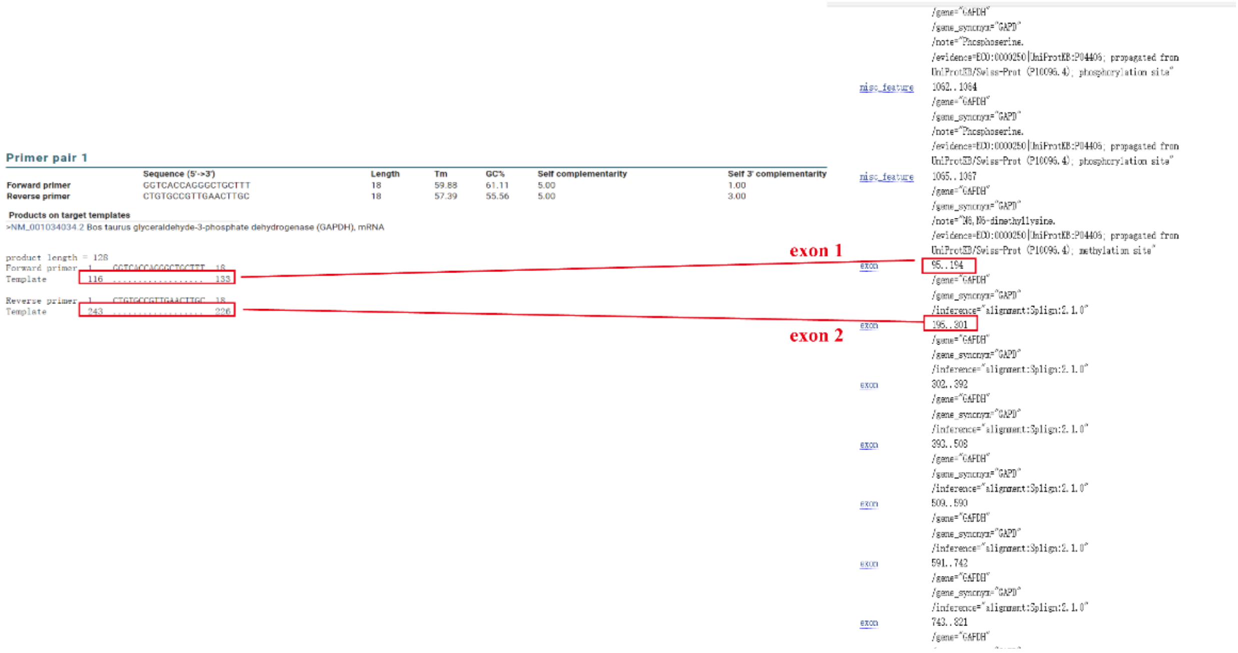

/gene_synonym="GAPD"
/inference="alignment:Splign:2.1.0"

4. Primer Amplification Efficiency Assessment

The standard curve is the most reliable and stable method for evaluating the efficiency of qPCR amplification. Using a series of diluted samples, Cq values were obtained by scaling up a standard qPCR procedure. Finally, draw a standard curve according to the log value of the concentration and the corresponding Cq value as follows:

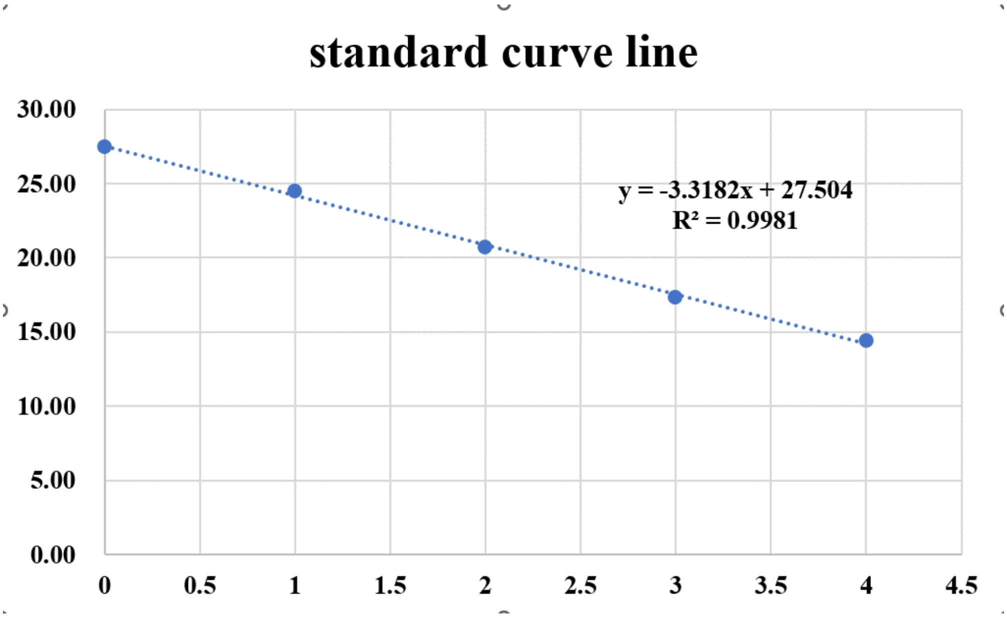

Amplification Efficiency (E) Calculation Formula: E=10$^{(-1/-3.3182)}$-1=100.15%

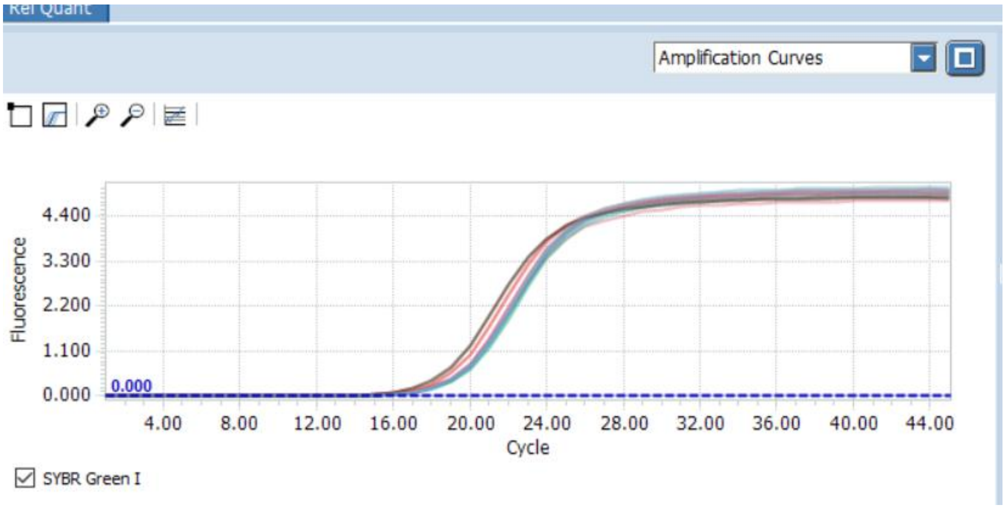

It is generally believed that the amplification efficiency should be between 90% ~110%, and the corresponding slope should be between -3.58 ~ -3.1. Our amplification efficiency result was 100.15%. Combined with the qPCR amplification curve, it can be seen that the primers we used have better amplification efficiency.
