# Supplementary material for: CTH/H2S Regulates LPS-Induced Inflammation through IL-8 Signaling in MAC-T Cells
Source: Int J Mol Sci. 2022 Oct 5;23(19):11822. doi: 10.3390/ijms231911822 (PMC9570289; doi:10.3390/ijms231911822)
Supplement: Supplementary file 1 [file ijms-23-11822-s001.zip › supplement data .pdf]

Supplement data

Table S1 Primers sequences used for qRT-PCR assay

Gene	Primer sequence (5'-3')	Fragment lengths	Tm/°C	Gene entry number
GAPDH	F: GGTCACCAGGGCTGCTTT<br>R: CTGTGCCGTTGAACTTGC	128bp	57.2	NM_001034034.2
CTH	F: AGGGCTCTCTTCAACATGCT<br>R: AAGCCCACTGAGAGTCGAAT	184bp	55.4	NM_001024567.1
CBS	F: CCGGAGAAGATGAGCACAGA<br>R: ATGTCATAGTGAGCGAGGGG	197bp	57.5	NM_001102000.2
IL-1β	F:TCAATAAAGTGCAAACTCCAGGACA<br>R: CTTGCACAAAGCTCATGCAGAA	133bp	56.3	NM_174093.1
IL-6	F: CCTTCACTCCATTCGCTGTCT<br>R: TCCTGATTTCCCTCATACTCG	391bp	57.6	NM_173923.2
IL-8	F: GCTGGCTGTTGCTCTCTTG<br>R: GGGTGGAAAGGTGTGGAATG	126bp	57.5	NM_173925.2
TNF-α	F: AAGCCTCAAGTAACAAGCCGGTAG<br>R: TCACACCGTTGGCCATGAG	108bp	59.6	NM_173966.3
TLR4	F: CATCATCTTCATCGTCCTG<br>R: ATCTGCTGTTCCTTCTGG	190bp	58.0	NM_174198.6

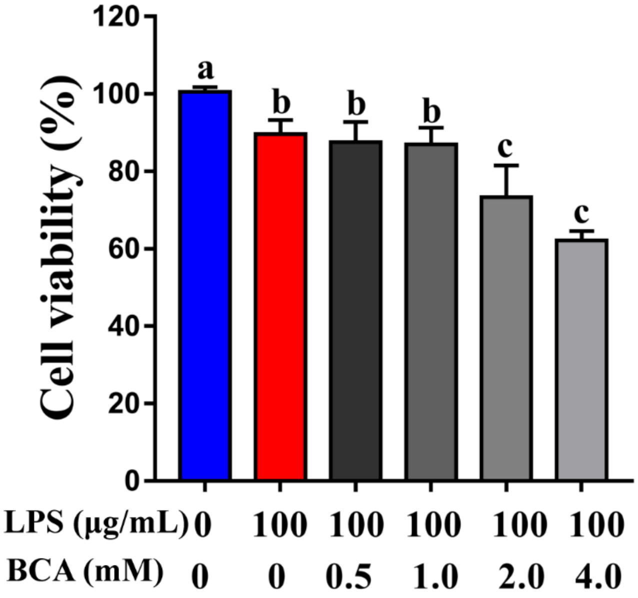

Fig. S1 Determination of BCA concentration for cell treatment in LPS-induced inflammatory model. Cell viability after LPS and/or BCA treatment with different concentration was monitored by CCK8 assay. **BCA:** β-cyano-L-Alanine. The different lowercase letters above the bars indicate a significant difference among different treatment groups ($P < 0.05$).
